# Spatio-temporal analysis of malaria vector density from baseline through intervention in a high transmission setting

**DOI:** 10.1186/s13071-016-1917-3

**Published:** 2016-12-12

**Authors:** Victor A. Alegana, Simon P. Kigozi, Joaniter Nankabirwa, Emmanuel Arinaitwe, Ruth Kigozi, Henry Mawejje, Maxwell Kilama, Nick W. Ruktanonchai, Corrine W. Ruktanonchai, Chris Drakeley, Steve W. Lindsay, Bryan Greenhouse, Moses R. Kamya, David L. Smith, Peter M. Atkinson, Grant Dorsey, Andrew J. Tatem

**Affiliations:** 1 WorldPop, Geography and Environment, University of Southampton, Southampton, UK; 2 Infectious Diseases Research Collaboration, Kampala, Uganda; 3 Department of Medicine, Makerere University College of Health Sciences, Kampala, Uganda; 4 Department of Medicine, San Francisco General Hospital, University of California, San Francisco, USA; 5 London School of Hygiene and Tropical Medicine, London, UK; 6 School of Biological and Biomedical Sciences, Durham University, Durham, UK; 7 Institute for Health Metrics and Evaluation, University of Washington, Seattle, USA; 8 Faculty of Science and Technology, Lancaster University, Lancaster, UK; 9 Flowminder Foundation, Stockholm, Sweden; 10 School of Geography, Archaeology and Palaeoecology, Queen’s University Belfast, Belfast, BT7 1NN Northern Ireland UK

**Keywords:** *Anopheles*, *Anopheles gambiae*, Indoor Residual Spraying, Malaria, Modelling

## Abstract

**Background:**

An increase in effective malaria control since 2000 has contributed to a decline in global malaria morbidity and mortality. Knowing when and how existing interventions could be combined to maximise their impact on malaria vectors can provide valuable information for national malaria control programs in different malaria endemic settings. Here, we assess the effect of indoor residual spraying on malaria vector densities in a high malaria endemic setting in eastern Uganda as part of a cohort study where the use of long-lasting insecticidal nets (LLINs) was high.

**Methods:**

*Anopheles* mosquitoes were sampled monthly using CDC light traps in 107 households selected randomly. Information on the use of malaria interventions in households was also gathered and recorded *via* a questionnaire. A Bayesian spatio-temporal model was then used to estimate mosquito densities adjusting for climatic and ecological variables and interventions.

**Results:**

*Anopheles gambiae* (*sensu lato*) were most abundant (89.1%; *n* = 119,008) compared to *An. funestus* (*sensu lato*) (10.1%, *n* = 13,529). Modelling results suggest that the addition of indoor residual spraying (bendiocarb) in an area with high coverage of permethrin-impregnated LLINs (99%) was associated with a major decrease in mosquito vector densities. The impact on *An. funestus* (*s.l.*) (Rate Ratio 0.1508; 97.5% CI: 0.0144–0.8495) was twice as great as for *An. gambiae* (*s.l*.) (RR 0.5941; 97.5% CI: 0.1432–0.8577).

**Conclusions:**

High coverage of active ingredients on walls depressed vector populations in intense malaria transmission settings. Sustained use of combined interventions would have a long-term impact on mosquito densities, limiting infectious biting.

**Electronic supplementary material:**

The online version of this article (doi:10.1186/s13071-016-1917-3) contains supplementary material, which is available to authorized users.

## Background

Since 2000, there has been a substantial decline in malaria morbidity and mortality globally [1], with prevalence of malaria infections in sub-Saharan Africa (SSA) dropping by a half, associated with the large-scale deployment of long-lasting insecticidal nets (LLINs), indoor residual spraying (IRS), and prompt and effective treatment with antimalarials [2, 3]. However, SSA still bears the largest *Plasmodium falciparum* malaria burden and malaria remains a public health problem [4]. This is partly because SSA has the most efficient malaria vector species [5, 6] namely; *Anopheles gambiae* (*s.s*.), *An. arabiensis* and other members of the *An. gambiae* complex; and *An. funestus* (*s.l*.) [7]. It is known that these vectors occur in sympatry across SSA [6]. They are all highly anthropophilic and prefer indoor biting [8]. *Anopheles arabiensis*, however, tends to be less endophagic, yet efficient enough to sustain transmission even if the other species were absent [9]. Vector control is central to the Global Technical Strategy (GTS) for malaria adopted in 2015 by the Global Malaria Programme (GMP) of the World Health Organization (WHO) [10]. Many countries are therefore adding indoor residual spraying (IRS) to the scale up of long lasting insecticidal nets (LLINs) for *P. falciparum* control [1, 11]. However, it remains unclear when and how best to combine IRS with LLINs [12], especially given an increasing documentation of pyrethroid resistance [13, 14].

There are a limited number of field-based studies assessing the combined impact of interventions on malaria vectors. Cluster randomized trial studies in Tanzania [15], and Benin [16] comparing IRS with bendiocarb plus LLINs *versus* LLINs alone remain inconclusive. The Tanzania study showed protective effect of IRS when combined with use of insecticide-treated nets (ITNs) compared to ITNs alone; however, the use of ITNs in the study population ranged from 53% at the start of the survey and declined to 36%. No additional protection was observed in the Benin study and also The Gambia trial [17], which compared LLINs to LLINs plus IRS with dichlorodiphenyltrichloroethane (DDT). Mathematical modelling techniques remain sensitive to the parameters used in the model [18, 19]. Observational non-randomised studies have also been inconclusive in terms of morbidity and mortality [20–24], and in most cases, it is difficult to assess the direct impact of adding IRS when LLIN or ITN use has not been scaled up in study populations. A multi-country analysis based on national representative household surveys from demographic health surveys (DHS) and malaria indicator surveys (MIS) conducted in 17 SSA countries reported mixed outcomes [20]. The use of combined intervention was protective for medium parasite rate in children 2 to 10 years old, (P*f*PR_2-10_ between 5 and 40%) and high malaria transmission settings (P*f*PR_2-10_ greater than 40%) but not for low transmission settings (less than 5% P*f*PR_2-10_). Given the heterogeneous distribution of mosquitoes, a longitudinal analysis based on field data could reveal how combinations of IRS and LLINs could impact malaria vectors.

Here, we build a geostatistical framework to estimate the spatial and temporal distribution and abundance of primary vectors as part of a longitudinal study in Nagongera sub-county, eastern Uganda from October 2011 to December 2015. The main objectives were to inform vector control strategies by investigating the direct effect of government initiated IRS on malaria vector species. Secondary objectives involved investigating their contemporary distribution and identifying extrinsic abiotic constraints (environmental and ecological covariates) associated with mosquito abundance.

## Methods

### Study area

The study was conducted in an extremely high stable malaria transmission intensity region of Eastern Uganda, south eastern border with Kenya, in Nagongera sub-county [25]. The area has an average altitude of 1,095 m above sea level and is dominated by subsistence farming (banana, maize and rice). The valleys are drained by east-west flowing streams joining the main river flowing to Lake Kyoga in central Uganda. The area in general experiences two rainy seasons averaging 1000–1500 mm of rainfall annually. The first wet and longer season is experienced early in the year between March to May and a shorter wet season is from October to November. The average day temperature is approximately 23 °C with the hottest months being January and February. Malaria transmission is characterised by two main peaks from March to June and November to December. The main malaria vectors in this area are the *An. gambiae* complex and the *An. funestus* complex [26, 27]. Full description of the study site and a map, is provided in Additional file 1.

### Entomology survey data

Entomological surveillance was conducted at household level as part of a cohort study described elsewhere [28, 29]. Briefly, a sampling frame of all the households in the area was established and 100 households selected randomly as part of dynamic cohort study targeting children aged between 6 months and 10 years. The first round of enrolment of households was conducted in August and September 2011, but, households could be dropped from the cohort if individuals moved out of the area. Thus, seven new households were selected in the second round of enrolment in 2013. Consent was obtained from the head of the household as part of the household survey. Mosquitoes were gathered once a month in selected households using the miniature CDC light traps (Model 512, John W. Hock Company, Gainesville, FL, USA). Traps were positioned at the foot end of the bed, next to a person sleeping under a LLIN with a light source placed approximately one meter above the floor [25]. Traps were placed at 1900 h and retrieved at 0700 h the following day. Most of the adult female *Anopheles* mosquitoes were identified using morphological characteristics (95%) with remaining few identified by polymerase chain reaction (PCR) technique. A comparison of the CDC light trap approach to other methods (i.e. the human-landing catches and pyrethrum spray catches) has already been undertaken elsewhere [25]. A summary of CDC light trap data is shown in section 1 of Additional file 1 and in Additional file 2.

### Climatic and environmental covariates

Plausible environmental covariates used for modelling vector densities were assembled. These included climatic (rainfall, temperature), ecological (enhanced vegetation index, EVI), topography (elevation), a proxy measure of urbanicity (night-time lights), Euclidean distance of the household to the water sources, and household density (defined as number of households within a 50 m radius of a selected houses). A generalized linear regression model implemented in the *bestglm* package in R was used to check for correlation between covariates that may result in multicollinearity [30]. Covariates (excluding intervention effects) were selected based on Bayesian information criterion (BIC) of most parsimonious non-spatial regression model. Thus, a model with lowest BIC was selected after covariates were regressed against the mosquito counts.

### Spatio-temporal analysis of mosquito vector density

A Bayesian hierarchical generalised mixed model with spatial and temporal effects was used to predict continuous maps of vector densities incorporating the above listed covariates and vector control interventions. An advantage of this approach is the ability to quantify uncertainty in the parameters of interest whilst including missing data points as NAs [31]. Mosquito counts for *An. gambiae* (*s.l*.) and *An. funestus* (*s.l*.), denoted as *y*_*ij*_; *i* = 1, … …, *n*; *j* = 1, … …, *m* where *i* is the household location, and *j* is the month were modelled as negative binomial [32, 33], with

1 $$ P\left({Y}_{ij}={y}_{ij}\right)=\frac{\varGamma \left(k+{y}_{ij}\right)}{\varGamma (k)\varGamma {y}_{ij}!}{p}^k{\left(1-p\right)}^{y_{ij}} $$

Where *Γ*(⋅) is a gamma function, with dispersion parameter *k*, and variance var(*y*_*ij*_) = *μ*_*ij*_ + *μ*_*ij*_^2^/*k* for mean *μ*_*ij*_. The outcome (mean vector density per household per night) for the general mixed effect regression model was of the form

2 $$ y\left({s}_i,{t}_j\right)={\beta}_0+{x}^T\left({s}_i,{t}_j\right){\beta}_i+{z}_i^T{\gamma}_j+ Season\left( mont{h}_j\right)+\nu \left({s}_i,{t}_j\right) $$

where *x* ^*T*^(*s*_*i*_, *t*_*j*_)*β*_*i*_ represented several set of covariate effects with *β*_*i*_ coefficients, *β*_0_ as intercept, *Ζ*_*i*_^*T*^ *γ*_*j*_ representing the random effects, and the last term *v*_*i*_ representing the spatial and temporal effect. Binary variables were included for each round of IRS at the household level. The government IRS campaign, using carbamate bendiocarb, was first conducted in the study area between December 2014 and February 2015 (round 1) followed by two rounds in June-July and December 2015. The IRS program was part of a national campaign that started in 2006 in epidemic regions (south western Uganda). In 2009, this was expanded to a further 10 districts in the northern parts of the country and to 14 high burden districts covering the mid-north, north east, mid-eastern and east central in 2014 including the study district (Tororo) [34]. The proportion of individuals sleeping under LLIN was included as a continuous variable. LLINs had been handed out to participating households at the start of the study in 2011 and through government mass campaigns in November 2013. A temporal, independent effect of month was included and modelled as an autoregressive process of first order *ξ*_*ij*_ ~ *N*(0, 1/*τ*(1 − *p* ^2^)) [35], with initial parameters selected based on a non-spatial time-series first order autoregressive model. Bayesian inference was performed using the integrated nested Laplace approximations (INLA) [36, 37] after setting prior distribution to model parameters (the intercept, covariates, spatial and temporal effects, and residual effects). INLA was used because of computational advantages compared to Markov chain Monte Carlo (MCMC) algorithms. This is because Gaussian Markov random fields (GMRFs) are used to represent the continuous domain Gaussian random field (GF) *via* stochastic partial differential equation (SPDE) approach resulting in computationally efficient matrices. Several plausible models were considered by varying the variable specification, i.e. most complex for all parameters (model 1) to least complex without spatio-temporal effects and interventions (model 5).

Model goodness-of-fits were assessed using a range of parameters including the deviance information criterion (DIC) and the marginal likelihood [38]. Validation was conducted by creating a subset dataset of *n* = 20 households selected randomly from the 107 households. Validation statistics included the correlation between the predicted and observed vector densities, and the root mean square error (RMSE). Full model specification details are provided in the supplementary.

## Results

**Fig. 1:**
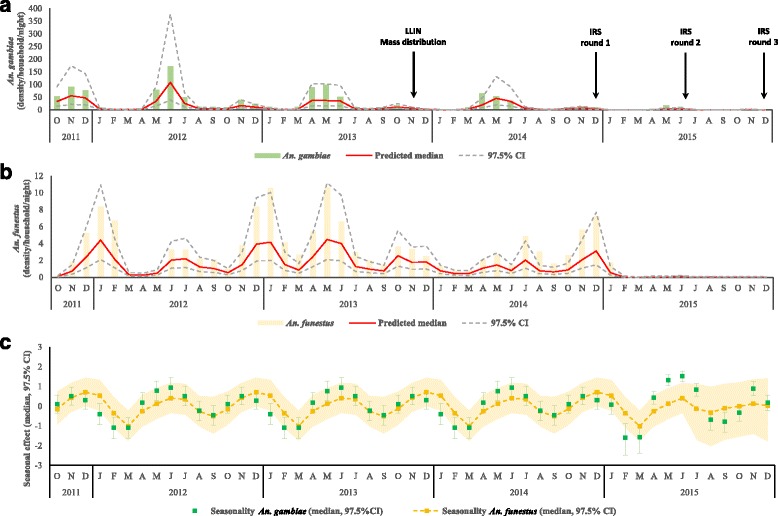
Median predicted average density of (**a**) *An. gambiae* (*s.l*.) and (**b**) *An. funestus* (*s.l*.) using household-level Bayesian spatio-temporal regression model with seasonal effects from September 2011 to December 2015. The bars represent the average observed counts while the solid line represents the median estimate. The dashed grey lines show the predicted 97.5% credible intervals. The median and quantiles are summarised quantities of posterior distribution. **c** The effect of season on both malaria vectors with bar plots for *An. gambiae* (*s.l*.) (97.5% credible interval) while the dashed line shows the median for *An. funestus* (*s.l*.) with the 97.5% credible interval shaded. *Abbreviations*: LLIN, long-lasting insecticidal nets; IRS, indoor residual spraying

**Fig. 2:**
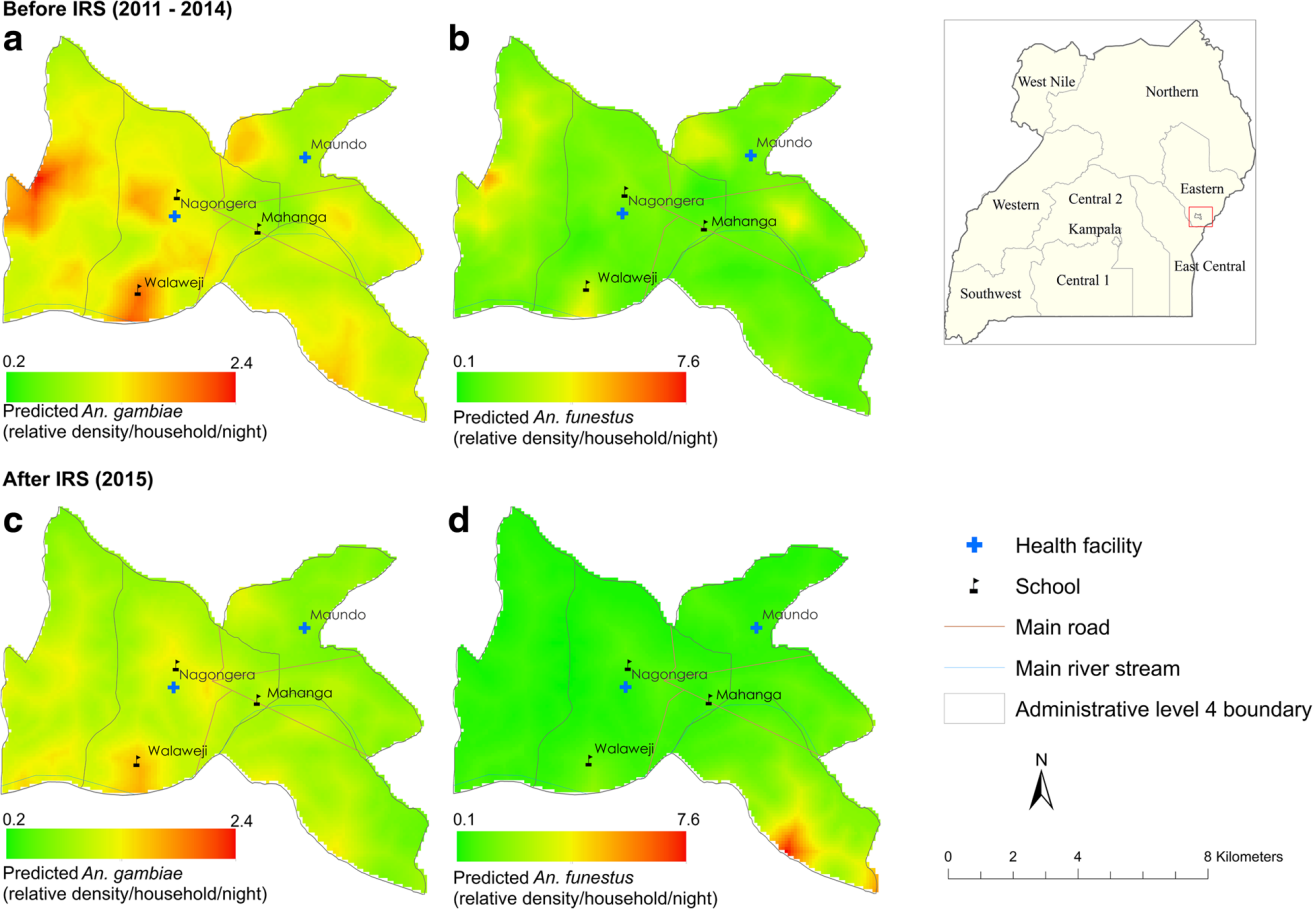
Map showing the study region in eastern Uganda. The four maps show the average predicted *An. gambiae* (*s.l*.) and *An. funestus* (*s.l*.) relative density per household per night, respectively, before (**a**, **b**) and after (**c**, **d**) IRS based on the Bayesian spatio-temporal model. The maps are normalised by the mean at 100 m spatial resolution. Thus, the highest predicted pre-IRS density for *An. gambiae* (*s.l*.) was 2.4 times as high as the mean compared to a prediction of 7.6 for *An. funestus (s.l.)*

A total of 133,528 adult female *Anopheles* mosquitoes were collected from 107 houses using CDC light traps between October 2011 and December 2015 (51 months). Of these, *An. gambiae* (*s.l*.) [including both *An. gambiae* (*s.s*.) and *An. arabiensis*, hereafter *An. gambiae* (*s.l*.)] were most abundant (119,008; 89.1%; 0.3% fed, followed by *An. funestus* (*s.l*.) (hereafter, *An. funestus*) (13,529; 10.1%; 1.0% fed). Since *An. gambiae* (*s.s*.) and *An. arabiensis* are not morphologically distinguishable, the proportion of each was examined molecularly and a small percentage (0.8%) were *An. arabiensis* [25]. Mosquito density was modelled by month and the spatio-temporal analysis showed highly heterogeneous and declining vector densities over time, with seasonal peaks twice in a year in May-June and November-December (Fig. 1). Figure 2 shows the predicted density maps pre-IRS (2011–2014) and post-IRS period (2015). Maps of spatial variance maps are included in the supplementary information.

Table 1 summarises the goodness-of-fit statistics for the five models based on a range of parameters. Comparisons based on combination of the deviance information criterion (DIC) and the marginal likelihood suggested that inclusion of spatial and temporal effects (i.e. models 1–3) improved the models, compared to excluding the spatial effects as in models 4 and 5. Where two DICs are similar, such as in models 1 and 2, this suggests that little information was gained by the more complex model and extra parametrisation. In terms of spatial parameters of the selected best fitting model (model 1), the spatial range (degree of similarity at household level) was 4 km for *An. gambiae* (*s.l*.) and 2.2 km for *An. funestus*. The estimated marginal variance was similar. Model 1 was therefore used for presenting subsequent results.

**Table 1:** Model fit and comparison using goodness-of-fit parameters for *An. gambiae* (*s.l*.) and *An. funestus* (*s.l*.). Model 1 included environmental and climatic variables; random effects (household level and seasonal); intervention use; and spatial and temporal effects. Model 5, the least complex, included only climatic variables and random effects. RMSE and correlation were based on a holdout validation dataset selected randomly (*n* = 20) out of a total 107 households

Vector species	Model	DIC	Model complexity	Marginal likelihood	RMSE	Correlation (Observed *vs* Predicted)
*An. gambiae* (*s.l*.)	Model 1	11083.56	122.05	-5743.87	1.1059	0.7963
	Model 2	11080.09	119.87	-5745.77	1.0565	0.7800
	Model 3	11082.78	120.42	-5757.49	1.0516	0.7777
	Model 4	11330.18	58.34	-5838.05	1.0883	0.7594
	Model 5	11329.69	56.37	-5827.67	1.0884	0.7592
*An. funestus (s.l.)*	Model 1	7188.35	134.51	-3783.64	0.9657	0.6937
	Model 2	7221.12	129.62	-3756.08	0.9615	0.6984
	Model 3	7194.15	119.54	-3764.33	0.9172	0.6930
	Model 4	7385.89	51.22	-3815.50	0.9259	0.6233
	Model 5	7385.90	50.89	-3806.26	0.9244	0.6252

*Abbreviations*: *DIC* Deviance information criterion, *RMSE* Root mean square error

### Effects of environmental and ecological factors on vector counts

Preliminary regression selected distance to water, elevation, night-time lights, EVI, temperature, rainfall, and household density for *An. gambiae* (*s.l*.). Only distance to water, elevation, EVI, rainfall, and household density were selected for *An. funestus*. EVI was positively correlated with precipitation (Spearman’s correlation coefficient of 0.6) but negatively correlated with temperature (Spearman’s correlation coefficient of 0.6). Table 2 displays the posterior mean estimates and 97.5% Bayesian credible intervals for the fixed effect parameters and hyperparameters for the spatio-temporal effect, month, and seasonality. As expected, rainfall and enhanced EVI had a positive (increasing) effect on mosquito densities. Distance to water, on the other hand, had a protective effect (10.6% decrease in adult vector abundance for every kilometre increase in distance to water body for *An. gambiae* (*s.l*.) and 15.8% for *An. funestus*). This is consistent with previous findings on declining mosquito densities with increasing distance from larval sources [39, 40]. The results for temperature and night-time lights were similar (protective effect) but not important. Day temperature varied between 19 °C and 40 °C and previous studies showed that higher temperatures (35 °C) decrease mosquito survival [41].

**Table 2:** Posterior rate ratio estimates and 97.5% credible interval (CI) for the best fitting model (Model 1) for *An. gambiae* (*s.l*.) and *An. funestus* (*s.l*.). The model includes all data range (2011–2015) and incorporates the effects of interventions. For the spatio-temporal specification, a parameter for the spatial range of influence is shown

Variable	*An. gambiae* (*s.l*.) Rate Ratio 97.5% CI				*An. funestus* (*s.l*.) Rate Ratio 97.5% CI			
	Mean	2.5%	50%	97.5%	Mean	2.5%	50%	97.5%
Distance to water (estimated in km)	0.8941	0.8520	0.8940	0.9367	0.8421	0.7819	0.8421	0.9064
Elevation (m above sea level)	0.9805	0.9162	0.9804	1.0520	1.0089	0.9294	1.0089	1.0955
Night-time light (intensity)	0.9749	0.9118	0.9748	1.0420	–	–	–	–
EVI	1.1434	1.0512	1.1433	1.2429	1.1606	1.0150	1.1605	1.3267
Temperature (estimated in °C)	0.9982	0.9166	0.9981	1.0868	–	–	–	–
Precipitation (mm)	1.0745	1.0185	1.0745	1.1331	0.9419	0.8438	0.9419	1.0475
Number of households within 50 m	0.9739	0.9153	0.9739	1.0371	0.9603	0.8885	0.9603	1.0384
IRS	0.5941	0.1432	0.5783	0.8577	0.1508	0.0144	0.1472	0.8495
LLIN	1.0026	0.9242	1.0025	1.0884	0.9770	0.8581	0.9769	1.1140
Spatial range (km) for Matérn covariance	4.6341	0.2772	4.6231	5.5876	2.2395	0.1996	2.2284	3.7029

*Abbreviations*: *EVI* Enhanced vegetation index, *IRS* Indoor residual spraying, *LLIN* Long lasting insecticide net

### Temporal effects

Figure 1 shows the modelled seasonal patterns associated with the two vector species. While the seasonal patterns were similar for the pre-IRS period (2011–2014), the uncertainty increased post-IRS in 2015 for both species. For *An. funestus*, model predictions showed dampened peaks in 2015 with a large range of uncertainty (the orange band). While similar patterns were predicted for *An. gambiae* (*s.l*.) across the years, the range of uncertainty between months increased slightly in 2015. Seasonal peaks and troughs established here provide guidance for targeting IRS before peak transmission [42], but, the dampened effect in 2015 (reduced seasonal peaks) highlights a reduced number of vectors observed at household level after spraying.

### Malaria intervention effects

At the start of the cohort study in 2011, all the enrolled households were provided with LLINs. Over 99% of cohort study participants reported sleeping under a LLIN the prior evening to the time of routine assessments, done every 3 months [43]. From cross-sectional surveys conducted in the surrounding community (non-cohort households), the proportion of households with at least one LLIN increased from 71.0% in January 2013 to 95.5% in January 2015, following a universal LLIN campaign conducted in November 2013. Modelling results show the community-wide deployment of LLINs (Table 2) did not reduce vector densities. In contrast, the government initiated IRS campaign implemented in December 2014 in the study area with rounds of bendiocarb sprayed approximately every 6 months, had an impact on vector densities. Nationally, IRS campaign in the high burden 14 districts in 2014 and 2015 (including Tororo district) achieved a coverage of 93.5% [34]. From the cohort, IRS was not done in only 5 houses in the first two rounds of IRS, and in 3 houses in the third round (excluding the 15 houses that dropped from the cohort before round 1 of the IRS). Modelling results showed that IRS was associated with a 40.6% decrease in mosquito densities for *An. gambiae* (*s.l*.)*,* compared to 84.9% for *An. funestus*, suggesting a two-fold greater effect on the latter species. Separate bioassays of vector susceptibility in Nagongera conducted in May 2014 showed 100% mortality rates for bendiocarb but less than 40% mortality for pyrethroids 24 h after exposure (unpublished data). This suggests a possible biological mechanism for the additional benefit of bendiocarb in settings with high coverage with pyrethroid-treated LLINs.

## Discussion

Vector control with large-scale deployment of LLINs and IRS are the major methods for malaria control in SSA [1]. However, as countries expand their malaria control programmes, there is a need to assess the impact of combined interventions on malaria vectors as well as health outcomes in routine conditions. The present study focused on entomological outcomes rather than clinical outcomes and the results provide an understanding of seasonal variation in the dominant malaria vectors as well as showing that IRS had an impact on malaria vectors in the setting of high LLIN coverage.

A longitudinal dataset was used to examine the temporal changes of malaria vector species in a high malaria transmission intensity setting. Such entomological surveillance data are rarely available to assess the impact of IRS on vector densities when there is a high coverage of pyrethroid-treated LLINs. The IRS effect was more evident on *An. funestus* species compared to *An. gambiae* (*s.l*.). While fewer *An. gambiae* (*s.l*.) were captured in 2015, IRS campaigns led to an almost total disappearance of *An. funestus* in the study cohort after two consecutive rounds (data shown in Additional files). Indeed, model estimates of the proportional effect of IRS were twice as high on *An. funestus* (RR 0.1508, 97.5% CI: 0.0144–0.8495) compared to *An. gambiae* (*s.l*.) (RR 0.5941, 97.5% CI: 0.1432–0.8577). In addition, *An. funestus* was only present in south-eastern parts of Nagongera after spraying when compared to the distribution before spraying rounds. This is consistent with other studies showing disproportionate impact of IRS on *An. funestus* [44]. Along with the quality of spraying (not quantified), the Uganda malaria control programme’s policy of conducting IRS after every 6 months, potentially increased its impact on the indoor-biting and resting anopheline mosquitoes in this area.

The results on declining vector density support findings on clinical outcomes elsewhere [28], and are also congruent with other entomological indicators such as the human biting rate [25]. Countries in SSA are changing to the use of carbamates and organophosphates, because of increasing evidence of DDT and pyrethroid resistance [13, 45–47]. One challenge of these insecticides is that they are more expensive than DDT and pyrethroids, which may lead to fewer houses being sprayed. However, it has been shown previously that the use of DDT or pyrethroids for IRS had less impact on morbidity compared to bendiocarb in other parts of Uganda [47, 48]. WHO tube bioassays on the effect of DDT and pyrethroids on *An. gambiae* (*s.l*.) in Nagongera showed moderate to high resistance (68, 24, and 37% mortality rate on *An. gambiae* (*s.l*.) for DDT, deltamethrin, and permethrin, respectively, unpublished data). Elsewhere in SSA, there were mixed results on the use of carbamates and DDT for IRS. For example, IRS with DDT did not offer an additional protective effect in communities with moderate malaria transmission and high coverage of LLINs in a cluster randomized trial in The Gambia where vector susceptibility was high [17]. A different observational study in the same country showed that carbamates and organophosphates were more effective compared to using DDT [45]. Additional empirical evidence from Benin also showed a high efficacy of bendiocarb [49]. On the contrary, there has been some evidence on the resistance of *An. gambiae* (*s.l*.) to carbamates particularly in West Africa in Côte d’Ivoire [50], Mali [46], and Burkina Faso [51]. This supports the need for continuous local monitoring of insecticide resistance and rotations or combinations of insecticides in areas with pyrethroid-treated LLINs as proposed by the WHO [52].

Modelling the seasonal cycles of malaria vectors provides useful information as to when IRS can be targeted for maximum efficacy and provide insights into transmission seasonality. From an operational perspective, the current WHO guidelines propose that the completion of indoor spraying should coincide with the build-up of vector density before the peak transmission season [42]. The modelled seasonality was similar for both vectors with bimodal peaks in June and December. This not only suggests that both species can be targeted for spraying at similar times or months of the year, but also shows that optimal targeting would aim at finishing spraying rounds before these peaks, such that there are fresh deposits of insecticides by May and December, respectively.

Rainfall and EVI were important drivers of vector density and seasonality while increasing distance from water sources was associated with lower mosquito density for both *An. gambiae* (*s.l*.) and *An. funestus*. It is important to use these abiotic factors in modelling adult vector densities as well as for spatial prediction. While past studies have shown that environmental variables are important drivers of seasonal patterns [6], the precise relationships and lag periods of these environmental variables are not well understood or generalizable for different malaria endemic settings [53]. In this study, the time-varying variables (rainfall, EVI and temperature) were not lagged. A separate analysis using a similar model specification but relaxing prior specification for covariates (i.e. using smoothing functions such as second-order random walks on continuous covariates) did not improve the goodness-of-fit. Thus, we opted to use these covariates with fixed prior specification [54]. In employing a longitudinal spatio-temporal analysis, the modelling framework addresses jointly the spatial and temporal correlation between sampled vector species at the household level [55]. A different model formulation, such as one that considers only spatial effects, may lead to different spatial variation and potentially mask the effect of time. Adult mosquito dispersal mechanisms can alter their spatial and temporal distribution substantially [56].

There are some additional caveats. The data used in this methodology focused on indoor biting malaria vectors and outdoor biting was not explored. Besides, the micro level effects of mosquito movement (i.e. indoor or outdoor) were not included in the analysis due to lack of empirical data. Although CDC light traps tend to sample indoor vectors [25], a recent study suggested that even the outdoor vectors attempt indoor biting [9]. A major finding by Kilama et al. [25] is the ethical suitability and acceptability of CDC light traps compared to the gold standard human landings or exit traps that can be logistically challenging, may be affected by collector biases, and the former also increases the risk of human infection. More importantly, different sampling strategies for the exophagic vectors should be considered [25], along with biotic interactions (competition and predation). Future studies should also consider an analysis of the influence of changing environment, interactions, and interventions on these exophagic vectors. However, while it is easier to assemble data on the environment, it is rather difficult to assemble longitudinal data on insecticide susceptibility or insecticide residual activity. This limits the form and type of analysis. Nonetheless, use of current environmental factors unearthed important seasonal patterns useful for vector control.

## Conclusions

The study demonstrated that there were major reductions in indoor-biting malaria vectors associated with IRS using bendiocarb when introduced in a community with high coverage and use of LLINs. The maps provide a spatial view of areas that can be targeted by spraying teams, for example, targeting the western parts of Nagongera. In Uganda, results elsewhere suggest that the use of bendiocarb complemented the high LLIN coverage [47, 48]. While these interventions are being scaled up nationally, insecticide resistance should be monitored continuously by the various national malaria control programmes. It is clear that the combination of vector control tools will not be sufficient to eliminate transmission from this area and that further interventions such as improved housing (ongoing trial in The Gambia [57]) are required to achieve this.

## Additional files

Additional file 1: Description of study site, covariates, and Bayesian geostatistical model. (DOC 1660 kb)
Additional file 2: Summary of average monthly mosquito counts gathered at household level for the study area. (DOC 80 kb)

## Abbreviations

BIC: Bayesian information criterion
CDC: Centre for Disease Control
DDT: Dichlorodiphenyltrichloroethane
DHS: Demographic health surveys
DIC: Deviance information criterion
EVI: Enhanced vegetation index
GF: Gaussian field
GMP: Global Malaria Programme
GMRF: Gaussian Markov random fields
GTS: Global technical strategy
INLA: Integrated nested Laplace approximation
IRS: Indoor residual spraying
LLIN: Long lasting insecticide net
MCMC: Markov chain Monte Carlo
MIS: Malaria indicator surveys
RMSE: Root mean square error
SPDE: Stochastic partial differential equation
SSA: Sub-Saharan Africa
WHO: World Health Organization
