# Supplementary material for: Spatio-temporal analysis of malaria vector density from baseline through intervention in a high transmission setting
Source: Parasit Vectors. 2016 Dec 12;9:637. doi: 10.1186/s13071-016-1917-3 (PMC5153881; doi:10.1186/s13071-016-1917-3)
Supplement: Additional file 1: — Description of study site, covariates, and Bayesian geostatistical model. (DOC 1660 kb) [file 13071_2016_1917_MOESM1_ESM.doc]

**Supplementary Information**

**Spatio-temporal analysis of malaria vector density from baseline through intervention in a high transmission setting**

Victor A Alegana, Simon P. Kigozi, Joaniter Nankabirwa, Emmanuel Arinaitwe, Ruth Kigozi, Henry Mawejje, Maxwell Kilama, Nick W. Ruktanonchai, Corrine W. Ruktanonchai, Chris Drakeley, Steve W. Lindsay, Bryan Greenhouse, Moses R. Kamya, David L. Smith, Peter M. Atkinson, Grant Dorsey, Andrew J. Tatem

# Abstract

**Background:** An increase in effective malaria control since 2000 has contributed to a decline in global malaria morbidity and mortality. Knowing when and how existing interventions could be combined to maximise their impact on malaria vectors can provide valuable information for national malaria control programs in different malaria endemic settings. Here, we assess the effect of indoor residual spraying on malaria vector densities in a high malaria endemic setting in eastern Uganda as part of a cohort study where the use of long-lasting insecticidal nets (LLINs) was high.

**Methods:** Anopheles mosquitoes were sampled monthly using CDC light traps in 107 households selected randomly. Information on the use of malaria interventions in households was also gathered and recorded via a questionnaire. A Bayesian spatio-temporal model was then used to estimate mosquito densities adjusting for climatic and ecological variables and interventions.

**Results:** *Anopheles gambiae* sensu lato were most abundant (89.1%; *n*=119,008) compared to *An. funestus* sensu lato (10.1%, *n*=13,529). Modelling results suggest that the addition of indoor residual spraying (bendiocarb) in an area with high coverage of permethrin-impregnated LLINs (99%) was associated with a major decrease in mosquito vector densities. The impact on *An. funestus* s.l. (Rate Ratio 0.1508 97.5% CI [0.0144 – 0.8495]) was twice as great as for *An. gambiae* s.l. (RR 0.5941 97.5% CI [0.1432 – 0.8577]).

**Conclusions:** High coverage of active ingredients on walls depressed vector populations in intense malaria transmission settings. Sustained use of combined interventions would have a long-term impact on mosquito densities, limiting infectious biting.

Table of Contents

[Abstract 1](#__RefHeading___Toc467675607)

[S1 Description of the study area, transmission intensity and dominant malaria vector species 3](#__RefHeading___Toc467675608)

[S1.1 Entomology survey data summary 3](#__RefHeading___Toc467675609)

[S2 Covariate processing and selection 4](#__RefHeading___Toc467675610)

[S2.1 Plausible environmental covariates for predicting adult mosquito vector densities 4](#__RefHeading___Toc467675611)

[S2.2 Data on use of LLINS and IRS 4](#__RefHeading___Toc467675612)

[S2.3 Covariate selection and test for multicollinearity 5](#__RefHeading___Toc467675613)

[S3 Non-spatial time series analysis 7](#__RefHeading___Toc467675614)

[S4 Model-based geostatistics for spatio-temporal estimation of mosquito vector density 8](#__RefHeading___Toc467675615)

[S4.1 Bayesian model specification 8](#__RefHeading___Toc467675616)

[S4.1 Bayesian model validation 11](#__RefHeading___Toc467675617)

[S4.2 Model uncertainty outputs 11](#__RefHeading___Toc467675618)

[S5 References 12](#__RefHeading___Toc467675619)

# S1 Description of the study area, transmission intensity and dominant malaria vector species

Map showing the study area in Eastern Uganda, south eastern border with Kenya, in Nagongera sub-county.


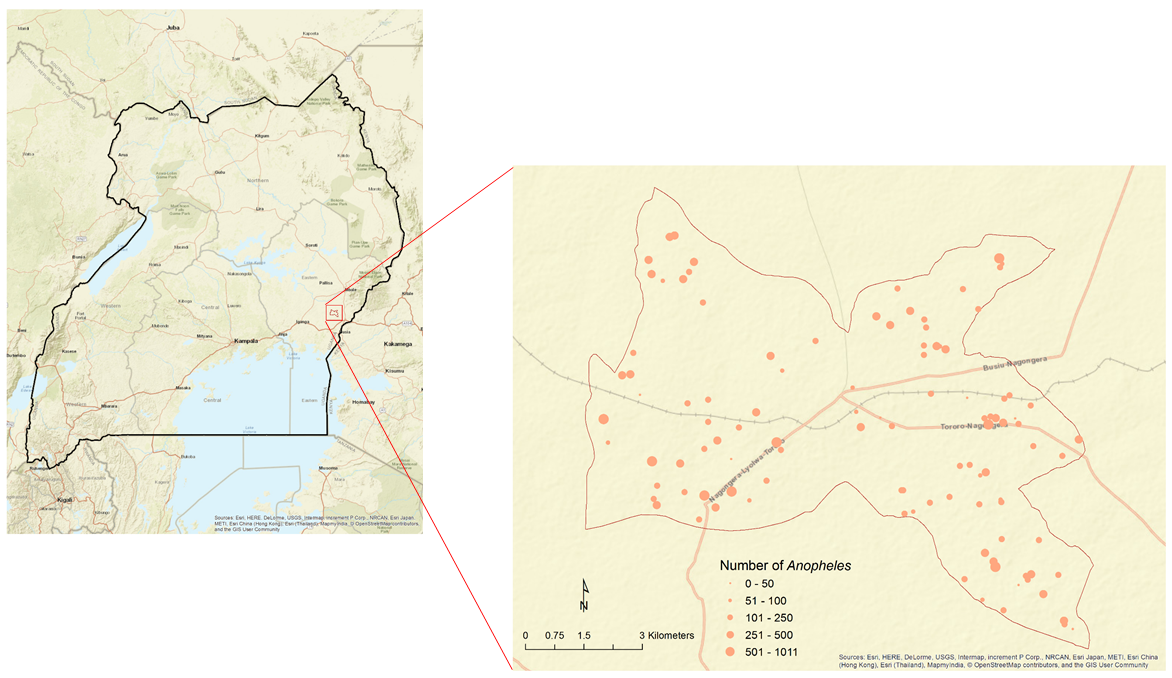


**Fig S1:** Base map of Nagongera sub-county with base map of major infrastructure of the area and average number of mosquitoes recorded at household (*n*=107).

## S1.1 Entomology survey data summary

A summary of data assembled for both mosquito species for the 51 month series is shown in table 1 below.

**Table S1.1**: Summary of average monthly mosquito counts gathered at household level for the study are

| **Year** |  |  | **Time (month)** | **Mean *An. gambiae* s.l.** | **Mean *An. funestus* s.l.** | **Difference in average *An. gambiae* s.l. recorded** | **Difference in average *An. funestus* s.l. recorded** |
| --- | --- | --- | --- | --- | --- | --- | --- |
| 2011 |  |  | 1 | 53.2 | 0.1 | - | - |
|  |  |  | 2 | 91.1 | 1.8 | 37.8 | 1.7 |
|  |  |  | 3 | 76.4 | 5.2 | -14.7 | 3.4 |
| 2012 |  |  | 4 | 9.9 | 8.4 | -66.4 | 3.1 |
|  |  |  | 5 | 4.1 | 6.7 | -5.8 | -1.6 |
|  |  |  | 6 | 2.0 | 0.6 | -2.1 | -6.2 |
|  |  |  | 7 | 6.5 | 0.3 | 4.5 | -0.2 |
|  |  |  | 8 | 78.6 | 0.7 | 72.1 | 0.4 |
|  |  |  | 9 | 171.8 | 3.3 | 93.2 | 2.6 |
|  |  |  | 10 | 48.8 | 3.3 | -123.0 | 0.0 |
|  |  |  | 11 | 11.4 | 2.2 | -37.5 | -1.1 |
|  |  |  | 12 | 12.4 | 2.1 | 1.0 | -0.1 |
|  |  |  | 13 | 9.9 | 1.1 | -2.5 | -1.0 |
|  |  |  | 14 | 38.6 | 3.8 | 28.7 | 2.7 |
|  |  |  | 15 | 23.7 | 8.4 | -14.9 | 4.6 |
| 2013 |  |  | 16 | 11.1 | 10.6 | -12.6 | 2.2 |
|  |  |  | 17 | 4.2 | 4.1 | -6.9 | -6.5 |
|  |  |  | 18 | 12.1 | 2.7 | 7.9 | -1.4 |
|  |  |  | 19 | 89.6 | 5.4 | 77.5 | 2.8 |
|  |  |  | 20 | 98.9 | 10.6 | 9.4 | 5.2 |
|  |  |  | 21 | 50.4 | 6.6 | -48.5 | -4.0 |
|  |  |  | 22 | 11.3 | 3.0 | -39.2 | -3.6 |
|  |  |  | 23 | 5.4 | 2.0 | -5.8 | -1.0 |
|  |  |  | 24 | 9.2 | 1.5 | 3.8 | -0.5 |
|  |  |  | 25 | 18.6 | 3.7 | 9.4 | 2.1 |
|  |  |  | 26 | 11.6 | 3.4 | -7.0 | -0.3 |
|  |  |  | 27 | 3.4 | 2.5 | -8.2 | -0.8 |
| 2014 |  |  | 28 | 1.1 | 1.4 | -2.2 | -1.2 |
|  |  |  | 29 | 1.4 | 0.8 | 0.3 | -0.6 |
|  |  |  | 30 | 8.7 | 0.9 | 7.3 | 0.1 |
|  |  |  | 31 | 65.7 | 2.1 | 57.0 | 1.2 |
|  |  |  | 32 | 52.7 | 2.7 | -13.0 | 0.6 |
|  |  |  | 33 | 35.4 | 1.4 | -17.3 | -1.3 |
|  |  |  | 34 | 12.3 | 4.9 | -23.1 | 3.4 |
|  |  |  | 35 | 4.5 | 3.1 | -7.8 | -1.8 |
|  |  |  | 36 | 1.1 | 1.6 | -3.5 | -1.5 |
|  |  |  | 37 | 9.9 | 2.6 | 8.8 | 1.0 |
|  |  |  | 38 | 13.8 | 5.7 | 3.9 | 3.0 |
|  |  |  | 39 | 9.3 | 7.3 | -4.6 | 1.6 |
| 2015 |  |  | 40 | 0.4 | 1.7 | -8.8 | -5.5 |
|  |  |  | 41 | 0.0 | 0.0 | -0.4 | -1.7 |
|  |  |  | 42 | 0.0 | 0.0 | 0.0 | 0.0 |
|  |  |  | 43 | 4.4 | 0.1 | 4.4 | 0.1 |
|  |  |  | 44 | 17.0 | 0.1 | 12.6 | 0.0 |
|  |  |  | 45 | 11.3 | 0.1 | -5.7 | 0.0 |
|  |  |  | 46 | 2.4 | 0.0 | -8.9 | -0.1 |
|  |  |  | 47 | 0.3 | 0.0 | -2.1 | 0.0 |
|  |  |  | 48 | 0.3 | 0.0 | 0.0 | 0.0 |
|  |  |  | 49 | 1.2 | 0.0 | 0.9 | 0.0 |
|  |  |  | 50 | 4.6 | 0.0 | 3.4 | 0.0 |
|  |  |  | 51 | 1.3 | 0.0 | -3.3 | 0.0 |

# S2 Covariate processing and selection

## S2.1 Plausible environmental covariates for predicting adult mosquito vector densities

Plausible environmental covariates used for modelling vector densities are summarised in Table S2.3. The monthly spatially gridded rainfall data at approximately 4 km resolution, from October 2011 to December 2015 (51 months), were assembled from the Tropical Applications of Meteorology using SATellite (TAMSAT) [1]. TAMSAT combines thermal infrared data from the geostationary Meteosat (meteorology) satellite acquired approximately every 15 minutes with ground based observations from over 4000 stations to predict rainfall amount. The gridded estimates were resampled to 1km spatial resolution to match with other spatial data. EVI and daily temperature were obtained from the MODerate-resolution Imaging Spectroradiometer (MODIS) imagery at a spatial resolution of 1 by 1 km (<http://modis.gsfc.nasa.gov/>) [2]. EVI is a measure of photosynthetic activity ranging from 0 (no vegetation) to 1 (complete vegetation). The night-time lights data, used as a proxy measure of urbanisation or urbanicity and human activity [3-5], were derived from Visible Infrared Imaging Radiometer Suite (VIIRS) [6]. Other static covariates included elevation, from the Shuttle Radar Topography Mission (SRTM) (http://srtm.usgs.gov/) and the Euclidean distance of the household to the river (stream) calculated using ESRI ArcGIS 10.3 Redlands, CA, spatial analysis tools.

## S2.2 Data on use of LLINS and IRS

Data on the use of LLINs and IRS were gathered as part of household surveys conducted between January and February every year. LLINs had been handed out to participating households at the start of the study in 2011 and through government mass campaigns in November 2013. The government IRS campaign, using carbamate bendiocarb, was first conducted between December 2014 and February 2015 (round 1) followed by two rounds in June-July and December 2015. An assumption was made regarding efficacious levels of pyrethroid concentration on LLINs of at least 2.5 years [7] with the first nets distributed in cohort households at the start of survey and then supplemented with government campaigns in 2013.

## S2.3 Covariate selection and test for multicollinearity

A generalized linear regression model implemented in the *bestglm* package in R using the leap algorithm was used to check for multicollinearity in the assembled covariates (Table 2.3) [8]. Covariates were selected based on Bayesian Information Criterion (BIC) of most parsimonious non-spatial regression approach described in the main text. Thus, a *glm* model with lowest BIC was selected after covariates were regressed against the mosquito counts. The coefficients and 95% confidence intervals of the best-fit covariates from the total-set analysis are shown in Table S2.1 below.

Table S2.1: Results of the covariate selection. Covariate selection analysis for Anopheles density modelling showing the regression coefficients and the *p*-values of the best-fit covariates

| **Covariates** | ***An. gambiae* s.l. (BIC-glm)** | | |  | ***An. funestus* (BIC-glm)** | | |
| --- | --- | --- | --- | --- | --- | --- | --- |
|  | **Coefficient** | **Standard error** | ***p*-value** |  | **Coefficient** | **Standard error** | ***p*-value** |
| Distance to water | -0.0678 | 0.0031 | < 0.001 |  | -0.1914 | 0.0101 | < 0.001 |
| Night-time lights (virs) | -0.0265 | 0.0033 | < 0.001 |  | - | - | - |
| Enhanced Vegetation Index (EVI) (mean) | 0.5404 | 0.0052 | < 0.001 |  | -0.0549 | 0.0130 | < 0.001 |
| Number of households within 50m | -0.0113 | 0.0035 | < 0.001 |  | 0.0425 | 0.0102 | < 0.001 |
| Precipitation | -0.2712 | 0.0044 | < 0.001 |  | -0.2938 | 0.0149 | < 0.001 |
| elevation | 0.0122 | 0.0032 | < 0.001 |  | -0.0353 | 0.0096 | < 0.001 |
| Temperature (day) | -0.4062 | 0.0048 | < 0.001 |  | - | - | - |

1. BIC used for model select ion criteria [8]
2. Blanks indicate covariates not selected

The analysis of correlation of the selected variable is shown in Table S2.2 with most variables showing a negative correlation.

**Table S2.2:** Pearson correlation of the selected variables

|  | Distance to water | Elevation | Enhanced vegetation Index (EVI) | Temperature | Number of households 50m | Night-time lights (virs) | Precipitation |
| --- | --- | --- | --- | --- | --- | --- | --- |
| Distance to water | 1.00 | -0.03 | 0.01 | 0.01 | -0.10 | 0.08 | 0.00 |
| Elevation | -0.03 | 1.00 | -0.06 | 0.03 | 0.08 | -0.31 | 0.02 |
| Enhanced vegetation Index (EVI) | 0.01 | -0.06 | 1.00 | -0.64 | -0.01 | 0.03 | 0.68 |
| Temperature | 0.01 | 0.03 | -0.64 | 1.00 | 0.00 | 0.01 | -0.48 |
| Number of households 50m | -0.10 | 0.08 | -0.01 | 0.00 | 1.00 | 0.26 | -0.01 |
| Night-time lights (virs) | 0.08 | -0.31 | 0.03 | 0.01 | 0.26 | 1.00 | -0.01 |
| Precipitation | 0.00 | 0.02 | 0.68 | -0.48 | -0.01 | -0.01 | 1.00 |

**Table S2.3:** Assembled plausible covariates for modelling vector density with associate descriptions.

| **Category** | **Covariate** | **Type** | **Description** | **Units/scale** | **Spatial resolution** | **Temporal resolution** | **Source** |
| --- | --- | --- | --- | --- | --- | --- | --- |
| Fixed effects | Precipitation | Continuous | Precipitation amount | Millimetres (mm) | ~ 4 km | 10 days | Tropical Applications of Meteorology using Satellite data (TAMSAT) [http://www.met.reading.ac.uk/tamsat/about/] |
|  | EVI | Continuous | Enhanced Vegetation Index | - | 1 km | Monthly | Moderate-resolution Imaging Spectroradiometer (MODIS) [http://modis.gsfc.nasa.gov/data/] |
|  | Temperature (day) | Continuous | Land surface temperature (day) | degrees Celsius | 1 km | 8 day composites averaged for month | Moderate-resolution Imaging Spectroradiometer (MODIS) [http://modis.gsfc.nasa.gov/data/] |
|  | Night-time light | Continuous | Proportion of observed stable light (night) | - | 1 km | - | VIIRS night-time lights (nano-Watts/(sqcm*sr)) [NOAA (http://ngdc.noaa.gov/eog/viirs.html)] |
|  | Distance to water/Rivers | Continuous | Euclidean distance to river | Kilometres | 1 km | - | Derived using ArcGIS and rivers shapefile of the area |
|  | Count of Households within 50m | Continuous | A count of households within 50 m radius | - | - | - | Derived from complete mapping of all households |
|  | Elevation | Continuous | Average height above sea level | Metres | 90 m | - | Shuttle Radar Topography Mission [http://srtm.usgs.gov/] |
| Random effects | Seasonality | Continuous | Time variable (monthly) | - | - | - | Household survey |
|  | Household unique identifier | Binary | Household unique id for each month | - | - | - | Household survey |
| Spatio-temporal | Latitude | Continuous | Latitude of the household | - | - | - | GPS |
|  | Longitude | Continuous | Longitude of the household | - | - | - | GPS |
|  | Month | Binary | Time variable | - | - | - | Household survey |
| Interventions | ITN | Continuous | Proportion of individuals that used an ITN night before survey /visit | - | - | - | Household survey |
|  | IRS | Binary | Household sprayed during IRS roll out | - | - | - | Household survey |

# S3 Non-spatial time series analysis

Non-spatial time series was conducted to test for stationarity in the outcome variable (mosquito counts) and validity of using autoregressive models for time series analysis. A Dickey-Fuller test was used for the former (testing for stationarity). For selection of autoregressive models, two models of first and second order were examined. Table 3.1 below shows results for both a test of stationarity and selection of autoregressive models.

**Table S3.1**: Non-spatial time series results for a test of stationarity (the Dickey-Fuller test) and autoregressive (ar(p) for p = 1, 2) models of first and second order for both *An. gambiae* s.l. and *An. funestus* s.l. For example, the resulting ar (1) model is of the form$x\left(t\right)=24.58(1-0.32)+0.32x\left(t-1\right)+w\left(t\right)$. The Dickey-Fuller test coefficient was significantly different from zero showing the data series was stationary. The autoregressive models were not different based on AIC even though the second order model had additional parameter and lower AIC.

|  | ***An. gambiae* complex** | |  | ***A. funestus* complex** | |
| --- | --- | --- | --- | --- | --- |
| Dickey-Fuller Test | -12.43 |  |  |  | -12.53 |
| Lag order | 15.00 |  |  |  | 15.00 |
| P-value | 0.01 |  |  |  | 0.01 |
|  | ar (1) | ar(2) |  | ar(1) | ar(2) |
| ar (1) parameter | 0.32 | 0.34 |  | 0.24 | 0.23 |
| ar (2) parameter | - | -0.07 |  | - | 0.06 |
| Mean | 24.58 | 24.58 |  | 2.70 | 2.70 |
| AIC | 43020.21 | 43003.32 |  | 25822.91 | 25809.93 |
| log-likelihood | -21507.10 | -21497.66 |  | -12908.45 | -12900.96 |

**Fig S2:** The autocorrelation function (ACF) and the partial autocorrelation function (PACF) for *An. gambiae* s.l and *An. funestus* s.l.. The ACF in both species show a rapid tail off in the first three to four lags suggesting an autoregressive process The PACF show similar result cutting off after first of second lags.

# S4 Model-based geostatistics for spatio-temporal estimation of mosquito vector density

## S4.1 Bayesian model specification

A Bayesian hierarchical space-time model implemented through implemented through an adapted stochastic partial differential equations (SPDE) approach and using the Integrated Nested Laplace Approximations (INLA) for Latent Gaussian Models (LGM) for inference [9, 10]. Bayesian inference was based on posterior distributions that combine data and appropriate prior knowledge (distributions) from model parameters via a likelihood function. The spatial effects introduced a measure of spatial autocorrelation in the model and thus, under Tobler’s first law of geography [11], households closer together in space would have similar vector densities compared to households that are further apart.

The outcome of interest was to model the *Anopheles* mosquito density in the 107 households. The mosquito counts were denoted as $y_{ij}$ $i=1,.....,n$; $j=1,.....,m$where $i$is the household location, and $j$is the month. There were 578 missing observations (approximately 10%) due to the dynamic nature of the cohort. Thus, seven households were replaced during the second round of enrolment in September 2013 and missing data points were treated as NAs rather than as model parameters. Missing data do not have an impact on the data-model likelihood. The counts for *An. gambiae* s.l. and *An. funestus* were modelled as negative binomial [12, 13] with

$P(Y_{ij}=y_{ij})=\frac{\Gamma\left(k+y_{ij}\right)}{\Gamma\left(k\right)\Gamma y_{ij}!}p^{k}(1-p)^{y_{ij}}$ 1

Where $\Gamma(\cdot)$is a gamma function, with dispersion parameter$k$, and variance $\mathrm{var}(y_{ij})=\mu_{ij}+\mu_{ij}^{2}/k$for mean$\mu_{ij}$. The outcome for the general mixed effect regression model was of the form [14]

$y(s_{i},t_{j})=\beta_{0}+x^{T}(s_{i},t_{j})\beta_{i}+z_{i}^{T}\gamma_{j}+Season(month_{j})+\nu(s_{i},t_{j})$ 2

where $x^{T}(s_{i},t_{j})\beta_{i}$ represented several set of covariate effects with $\beta_{i}$ coefficients, $\beta_{0}$ is the intercept while $Z_{i}^{T}\gamma_{j}$represents the additive terms of random effects with the last term $\nu(s_{i},t_{j})$ representing the spatial and temporal effect. Binary variables were included for each round of IRS at the household level. The proportion of individuals sleeping under LLIN was included as a continuous variable. A temporal, independent effect of month was included and modelled as an autoregressive process of first order $\xi_{ij}\sim N(0,1/\tau(1-\rho^{2}))$ [15, 16] with initial parameters selected based on a non-spatial time-series first order autoregressive model (See S3)**.**

The spatial effect (spatial covariance) was modelled using the stochastic partial differential equation (SPDE) approach [17-19]. For the combined spatio-temporal effect, an SPDE of the form

$\left(k(s)^{2}-\Delta\right)^{\alpha/2}\left(\tau(s)x(s,t)\right)=w(s,t)$; $\alpha=\nu+d/2$;$k>0$,$v>0$ 3

with Matérn spatial covariance was used; where $s$ denotes the spatial domain (household location); $t$the time domain; $\left(k(s)^{2}-\Delta\right)^{\alpha/2}$is a differential operator; $k$ is the scaling parameter; $\Delta$ is the Laplacian; $\alpha$controls the smoothness of the realization;$\tau$controls the variance, $x(s,t)$is the weight vector; and $w(s,t)$on the right hand side is the Gaussian white noise. The Matérn covariance of the form

$C(\cdot)=\frac{\sigma^{2}}{2^{v-1}\Gamma(v)}\left(a||h||\right)^{v}k_{v}\left(a||h||\right)$ 4

was used, where is the modified Bessel function of the second order kind and is the Euclidean distance while is the marginal spatial variance. $a$ is the scaling parameter, with $a>0$, while $\nu$ is the Matérn smoothness parameter as defined above and is linked to the spatial variance $\sigma^{2}$ through $\alpha=\nu+d/2$where *d* is the spatial dimension. The spatial variance is then given by$\sigma^{2}\approx1/4\pi k(s)^{2}\tau(s)^{2}$. The model specification relates the scaling parameter to the spatial range $r$ via $r\approx\sqrt{8}/k(s)$ with an initial parameter approximation $r=1/5$ for the area of study.

The joint spatial and temporal specification generates a separable variance-covariance matrix of the form $C\left(\cdot\right)=C(s)\cdot C(t)$ with precision where is the precision in the spatial domain and in temporal domain [20]. Seasonal variation with periodicity of 12 months for *n*=51 months was incorporated to isolate the effect of month-to-month changes in vector density using a simple Gaussian vector specification $\lambda_{j}\sim N(0,1/\tau_{\lambda_{j}})$ with a gamma prior specification on the precision parameter$\tau\sim Ga(1,5e-05)$. The length of periodicity defines the smoothing effects on month-to-month variation in response. For example, fitting the seasonal component with a shorter periodicity of six months increases jumps in data [21, 22]. We found an optimal setting of 12 months suitable for seasonality random effect. Preliminary analyses decomposed the seasonality terms into seasonal effects and a linear trend parameter. The linear trend was, however, removed in final analysis because it was not significant. The Bayesian specification was completed by assigning zero mean Gaussian prior distributions for the regression parameters such that$\beta_{j}\sim N(0,1/\tau_{\beta_{j}})$, the hyper-parameters for the temporal effect (month), a household random effect (assumed to be independent and identically distributed)$\tau\sim Ga(1,5e-05)$. The initial parameters for autoregressive models were based on the non-spatial time series analysis (section 3 of the supplementary information). With the number, size and complexity of the models to be considered (Table S4.1), Bayesian inference was achieved using approximate solutions by using the Integrated Nested Laplace Approximation (INLA) approach [10, 16]. Parameters of interest in the posterior distribution included the mean, median, the credible intervals of average counts of *An. gambiae* s.l. and *An. funestus*. Model representation is graphically shown in Figure S3 below.

**Fig S3:** Graphical representation of the mode showing parameter space

of spatio-temporal regression.

## S4.1 Bayesian model validation

Several plausible models were considered by varying the variable specification (i.e. fixed parameters, random, spatio-temporal effects, and controlling for the effects of interventions).

Validation was conducted by creating a subset dataset of *n*=20 households selected randomly from the 107 households. Validation statistics included the correlation between the predicted and observed vector densities, the root mean square error (RMSE), and proportion of variance unexplained by the different model specifications. Predictive performance was assessed using leave-one out cross-validation procedures based on the predictive distribution [23].

**Table S4.1: Model consideration and specification for longitudinal analysis of mosquito vector density.**

|  | Fixed Parameters including model Intercept (Covariates) Precipitation, Temperature, Vegetation, Night-time lights, Elevation, distance to water, and number of households within 50m | Random effects (Household effects, Monthly (first order ARIMA), Seasonality effect with period of 12 months) | Interventions (IRS and LLIN use (Proportion of individuals sleeping under an LLIN) | Bayesian Spatio-temporal effects via SPDE approach with a stationary Matérn covariance specification |
| --- | --- | --- | --- | --- |
| Model 1 | x | x | x | x |
| Model 2 | x | x | - | x |
| Model 3 | x | - | x | x |
| Model 4 | x | x | x | - |
| Model 5 | x | x | - | - |

*Model 3 excluded only a household random effect. The month and seasonality effect were included.

*LLIN use was used as a continuous variable defined as proportion of people in the household using an insecticide treated net

## S4.2 Model uncertainty outputs


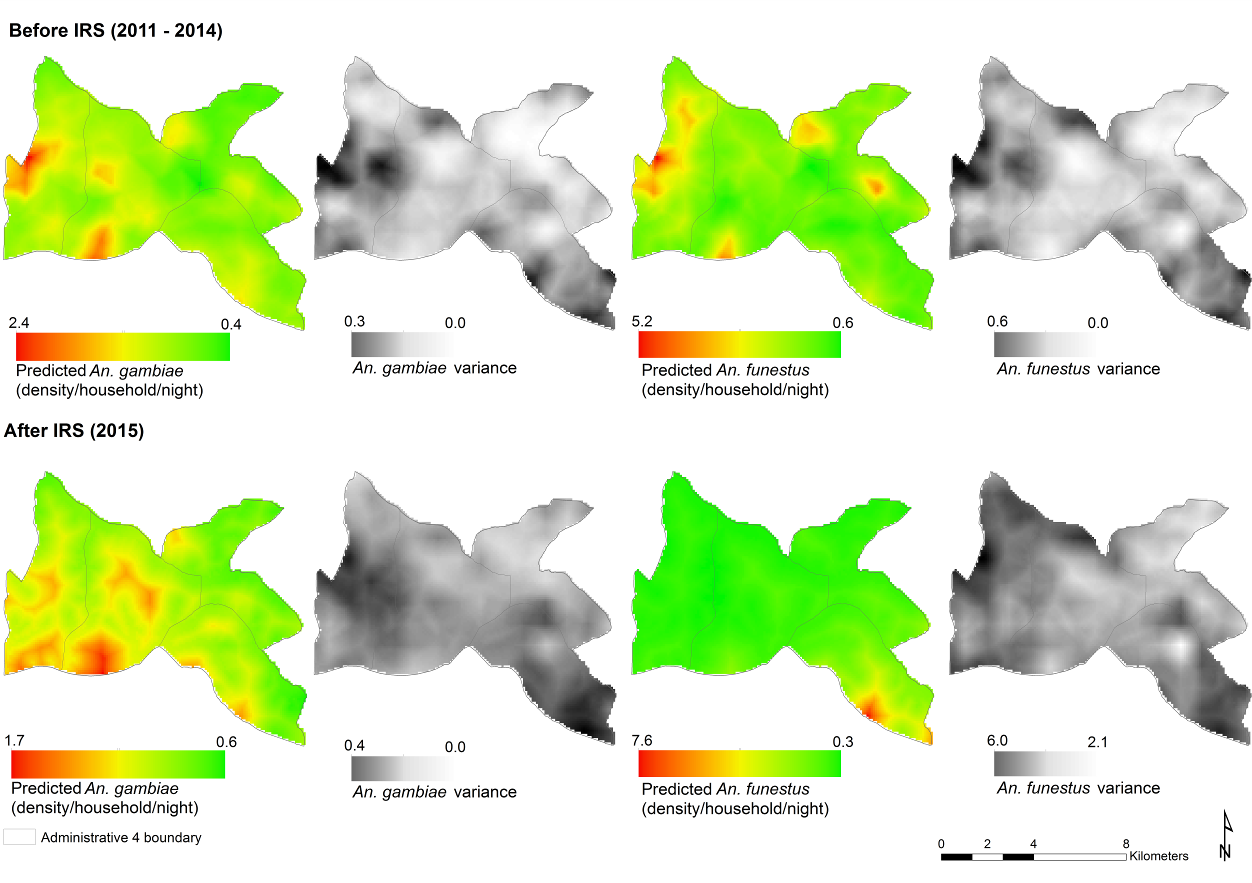


**Fig S4**: Maps showing the average predicted *An. gambiae* and *An. funestus* densities respectively at 100 m spatial resolution and the variance.

# S5 References
