# Supplementary material for: Spatio-temporal analysis of malaria vector density from baseline through intervention in a high transmission setting
Source: Parasit Vectors. 2016 Dec 12;9:637. doi: 10.1186/s13071-016-1917-3 (PMC5153881; doi:10.1186/s13071-016-1917-3)
Supplement: Additional file 2: — Summary of average monthly mosquito counts gathered at household level for the study area. (DOC 80 kb) [file 13071_2016_1917_MOESM2_ESM.doc]

**Table S1**: Summary of average monthly mosquito counts gathered at household level for the study area

| **Year** |  |  | **Time (month)** | **Mean *An. gambiae* (s.l.)** | **Mean *An. funestus (*s.l.)** | **Difference in average *An. gambiae* (s.l.) recorded** | **Difference in average *An. funestus* (s.l.) recorded** |
| --- | --- | --- | --- | --- | --- | --- | --- |
| 2011 |  |  | 1 | 53.2 | 0.1 | - | - |
|  |  |  | 2 | 91.1 | 1.8 | 37.8 | 1.7 |
|  |  |  | 3 | 76.4 | 5.2 | -14.7 | 3.4 |
| 2012 |  |  | 4 | 9.9 | 8.4 | -66.4 | 3.1 |
|  |  |  | 5 | 4.1 | 6.7 | -5.8 | -1.6 |
|  |  |  | 6 | 2.0 | 0.6 | -2.1 | -6.2 |
|  |  |  | 7 | 6.5 | 0.3 | 4.5 | -0.2 |
|  |  |  | 8 | 78.6 | 0.7 | 72.1 | 0.4 |
|  |  |  | 9 | 171.8 | 3.3 | 93.2 | 2.6 |
|  |  |  | 10 | 48.8 | 3.3 | -123.0 | 0.0 |
|  |  |  | 11 | 11.4 | 2.2 | -37.5 | -1.1 |
|  |  |  | 12 | 12.4 | 2.1 | 1.0 | -0.1 |
|  |  |  | 13 | 9.9 | 1.1 | -2.5 | -1.0 |
|  |  |  | 14 | 38.6 | 3.8 | 28.7 | 2.7 |
|  |  |  | 15 | 23.7 | 8.4 | -14.9 | 4.6 |
| 2013 |  |  | 16 | 11.1 | 10.6 | -12.6 | 2.2 |
|  |  |  | 17 | 4.2 | 4.1 | -6.9 | -6.5 |
|  |  |  | 18 | 12.1 | 2.7 | 7.9 | -1.4 |
|  |  |  | 19 | 89.6 | 5.4 | 77.5 | 2.8 |
|  |  |  | 20 | 98.9 | 10.6 | 9.4 | 5.2 |
|  |  |  | 21 | 50.4 | 6.6 | -48.5 | -4.0 |
|  |  |  | 22 | 11.3 | 3.0 | -39.2 | -3.6 |
|  |  |  | 23 | 5.4 | 2.0 | -5.8 | -1.0 |
|  |  |  | 24 | 9.2 | 1.5 | 3.8 | -0.5 |
|  |  |  | 25 | 18.6 | 3.7 | 9.4 | 2.1 |
|  |  |  | 26 | 11.6 | 3.4 | -7.0 | -0.3 |
|  |  |  | 27 | 3.4 | 2.5 | -8.2 | -0.8 |
| 2014 |  |  | 28 | 1.1 | 1.4 | -2.2 | -1.2 |
|  |  |  | 29 | 1.4 | 0.8 | 0.3 | -0.6 |
|  |  |  | 30 | 8.7 | 0.9 | 7.3 | 0.1 |
|  |  |  | 31 | 65.7 | 2.1 | 57.0 | 1.2 |
|  |  |  | 32 | 52.7 | 2.7 | -13.0 | 0.6 |
|  |  |  | 33 | 35.4 | 1.4 | -17.3 | -1.3 |
|  |  |  | 34 | 12.3 | 4.9 | -23.1 | 3.4 |
|  |  |  | 35 | 4.5 | 3.1 | -7.8 | -1.8 |
|  |  |  | 36 | 1.1 | 1.6 | -3.5 | -1.5 |
|  |  |  | 37 | 9.9 | 2.6 | 8.8 | 1.0 |
|  |  |  | 38 | 13.8 | 5.7 | 3.9 | 3.0 |
|  |  |  | 39 | 9.3 | 7.3 | -4.6 | 1.6 |
| 2015 |  |  | 40 | 0.4 | 1.7 | -8.8 | -5.5 |
|  |  |  | 41 | 0.0 | 0.0 | -0.4 | -1.7 |
|  |  |  | 42 | 0.0 | 0.0 | 0.0 | 0.0 |
|  |  |  | 43 | 4.4 | 0.1 | 4.4 | 0.1 |
|  |  |  | 44 | 17.0 | 0.1 | 12.6 | 0.0 |
|  |  |  | 45 | 11.3 | 0.1 | -5.7 | 0.0 |
|  |  |  | 46 | 2.4 | 0.0 | -8.9 | -0.1 |
|  |  |  | 47 | 0.3 | 0.0 | -2.1 | 0.0 |
|  |  |  | 48 | 0.3 | 0.0 | 0.0 | 0.0 |
|  |  |  | 49 | 1.2 | 0.0 | 0.9 | 0.0 |
|  |  |  | 50 | 4.6 | 0.0 | 3.4 | 0.0 |
|  |  |  | 51 | 1.3 | 0.0 | -3.3 | 0.0 |
